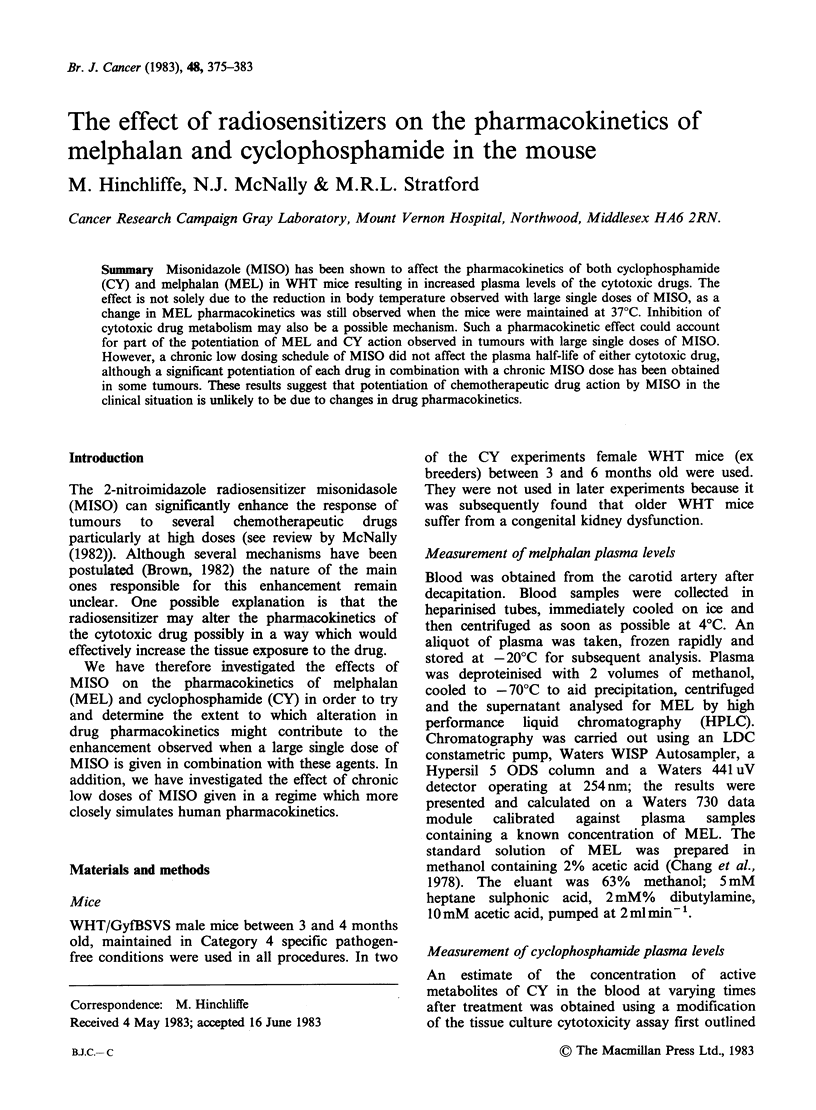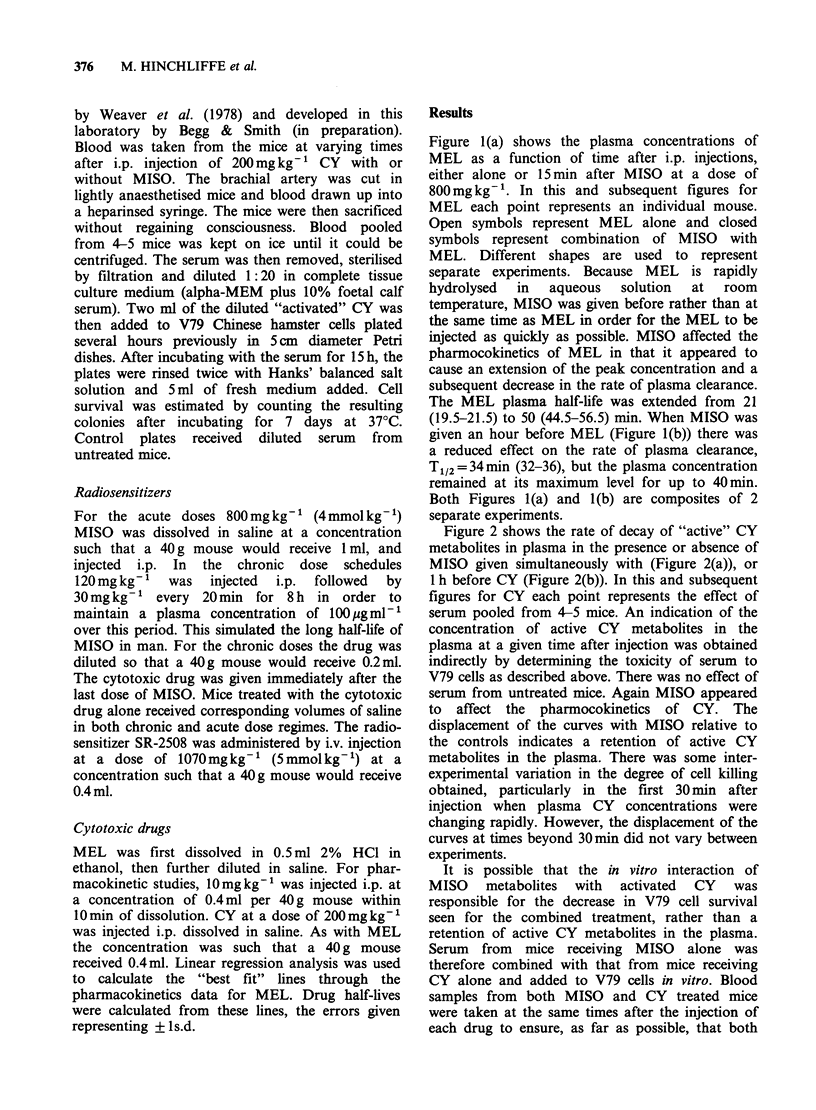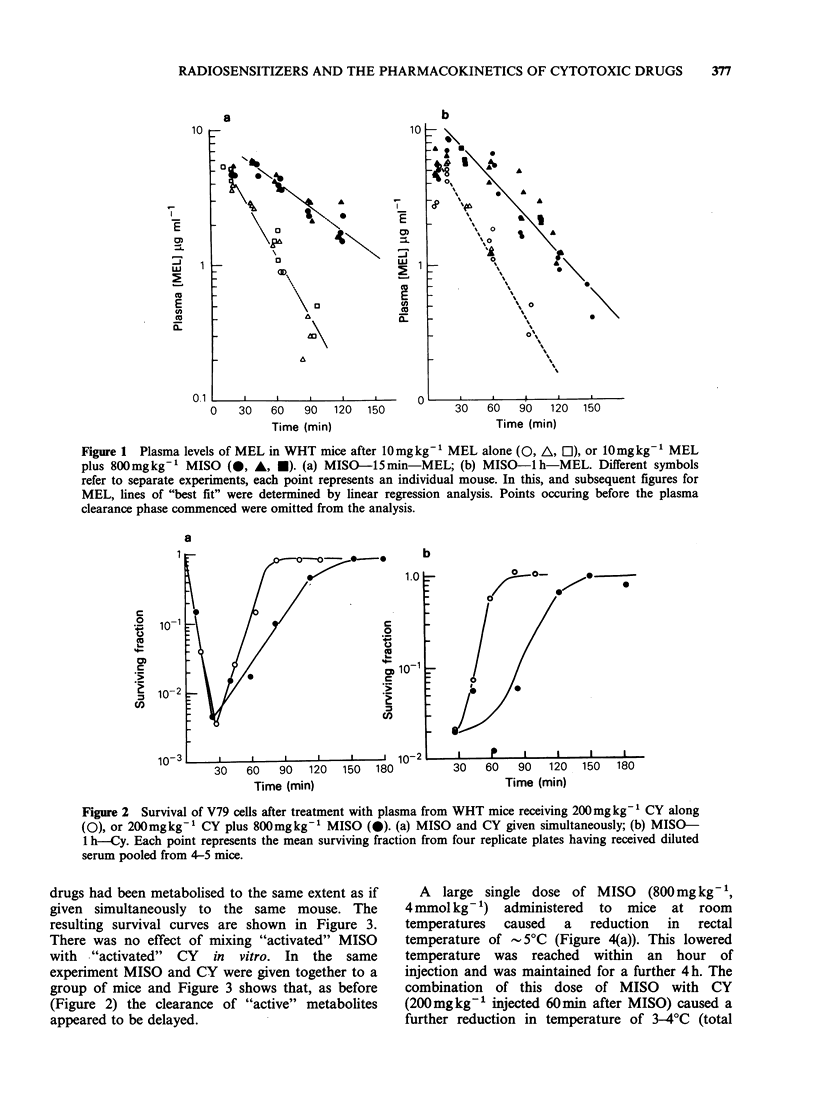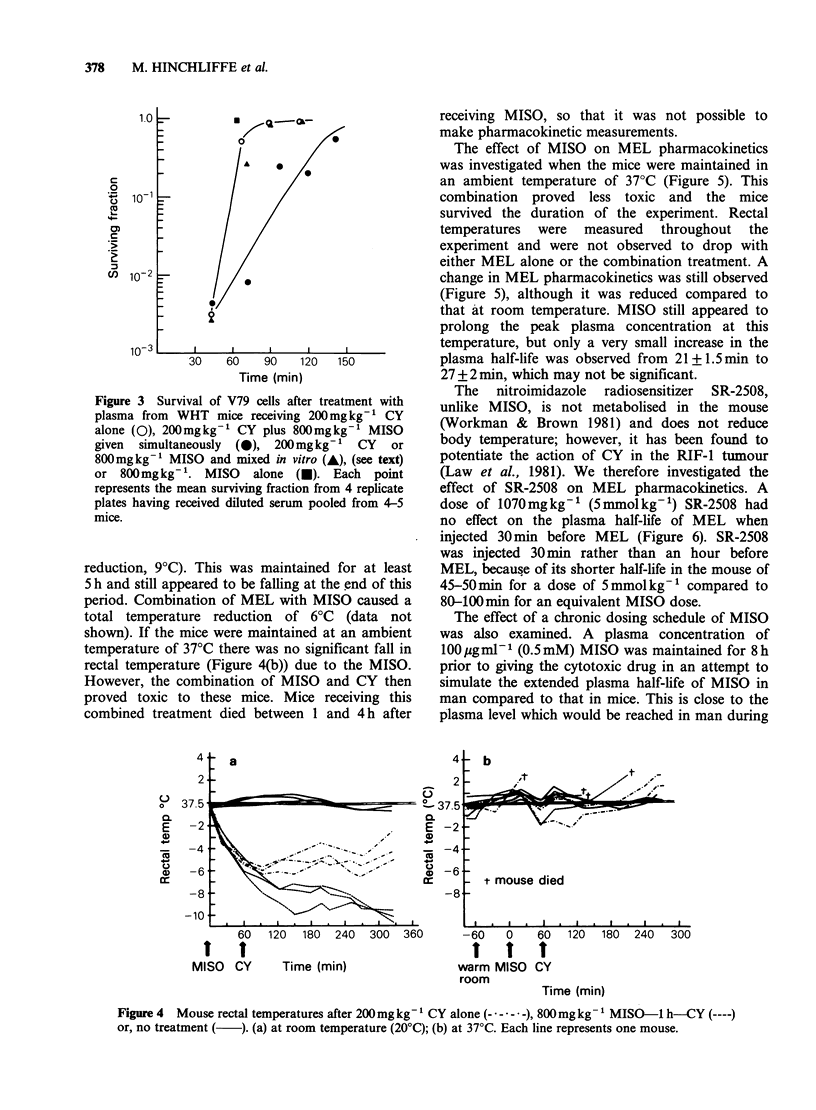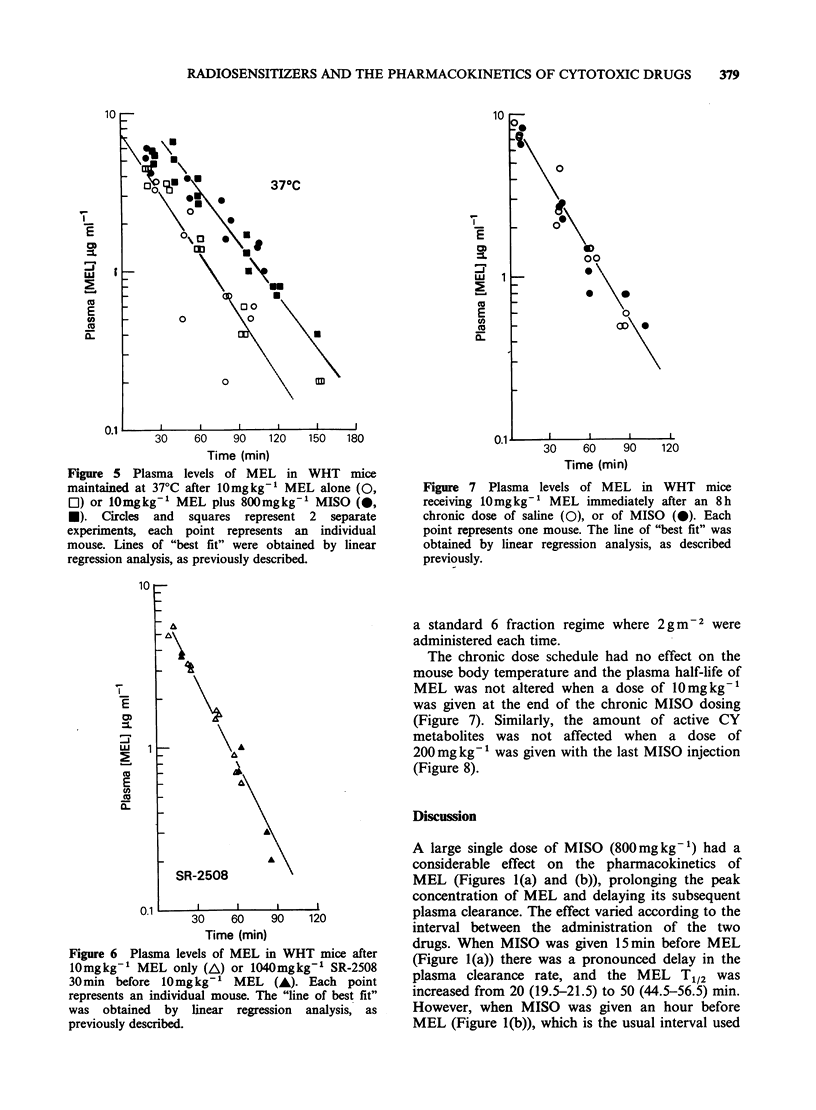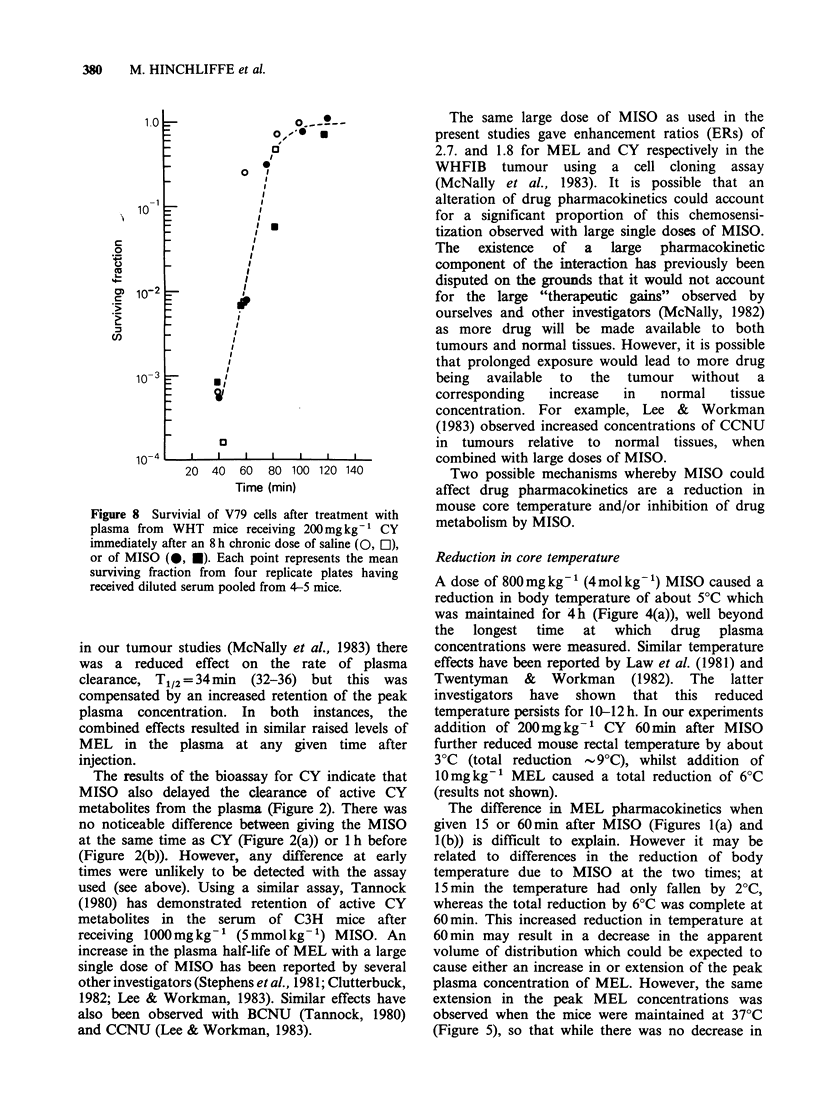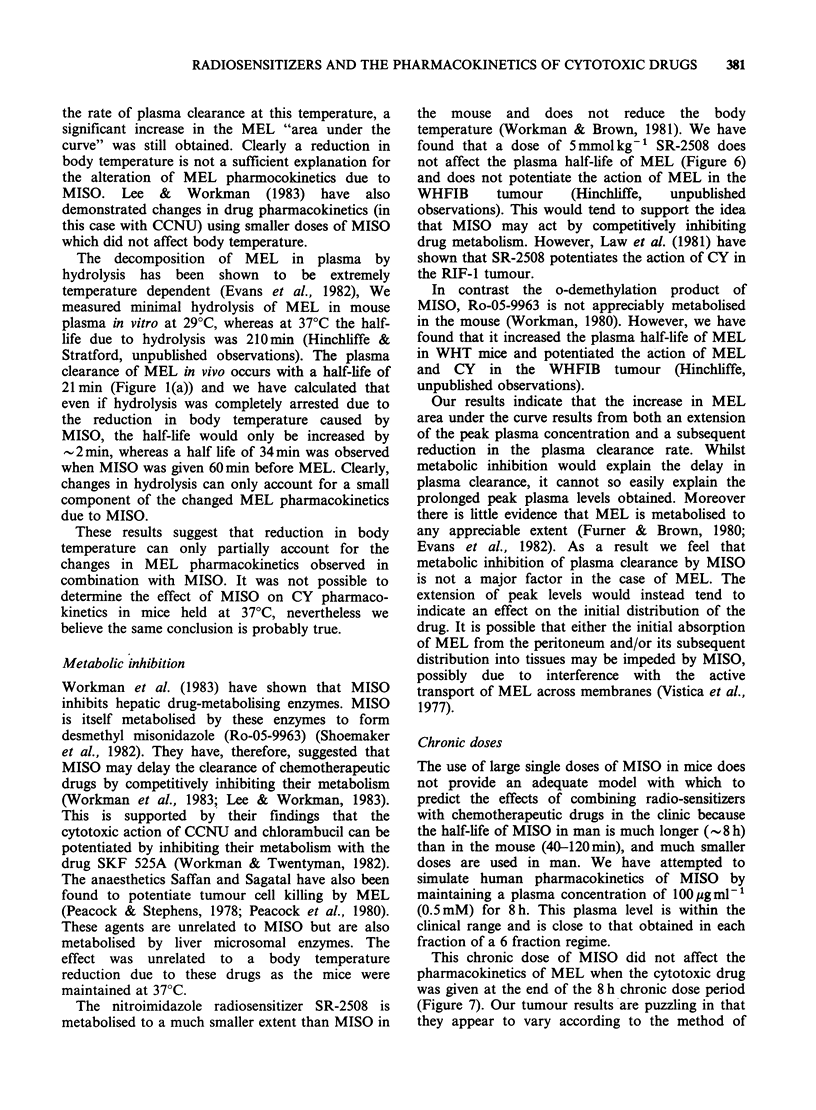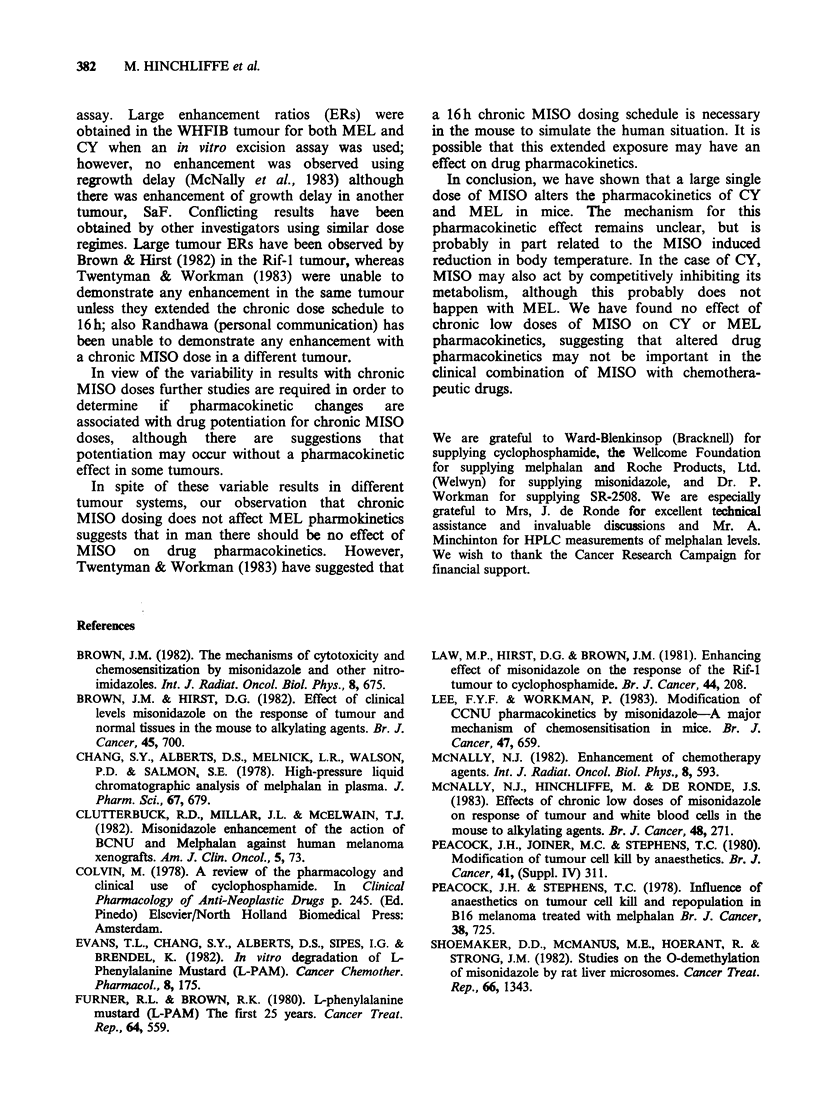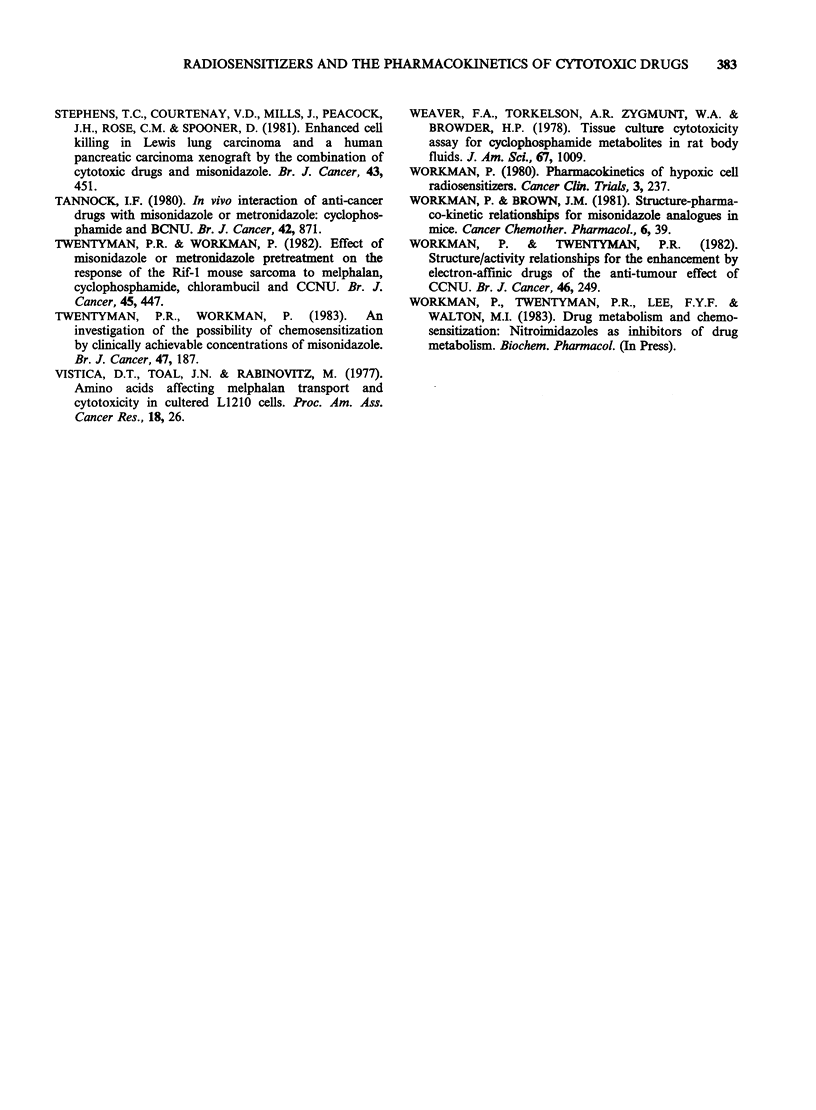# The effect of radiosensitizers on the pharmacokinetics of melphalan and cyclophosphamide in the mouse.

**DOI:** 10.1038/bjc.1983.202

**Published:** 1983-09

**Authors:** M. Hinchliffe, N. J. McNally, M. R. Stratford

## Abstract

Misonidazole (MISO) has been shown to affect the pharmacokinetics of both cyclophosphamide (CY) and melphalan (MEL) in WHT mice resulting in increased plasma levels of the cytotoxic drugs. The effect is not solely due to the reduction in body temperature observed with large single doses of MISO, as a change in MEL pharmacokinetics was still observed when the mice were maintained at 37 degrees C. Inhibition of cytotoxic drug metabolism may also be a possible mechanism. Such a pharmacokinetic effect could account for part of the potentiation of MEL and CY action observed in tumours with large single doses of MISO. However, a chronic low dosing schedule of MISO did not affect the plasma half-life of either cytotoxic drug, although a significant potentiation of each drug in combination with a chronic MISO dose has been obtained in some tumours. These results suggest that potentiation of chemotherapeutic drug action by MISO in the clinical situation is unlikely to be due to changes in drug pharmacokinetics.


*Br. J. Cancer* (1983), **48**, 375–383

### The effect of radiosensitizers on the pharmacokinetics of melphalan and cyclophosphamide in the mouse

M. Hinchliffe, N.J. McNally & M.R.L. Stratford

*Cancer Research Campaign Gray Laboratory, Mount Vernon Hospital, Northwood, Middlesex HA6 2RN.*

**Summary** Misonidazole (MISO) has been shown to affect the pharmacokinetics of both cyclophosphamide (CY) and melphalan (MEL) in WHT mice resulting in increased plasma levels of the cytotoxic drugs. The effect is not solely due to the reduction in body temperature observed with large single doses of MISO, as a change in MEL pharmacokinetics was still observed when the mice were maintained at 37°C. Inhibition of cytotoxic drug metabolism may also be a possible mechanism. Such a pharmacokinetic effect could account for part of the potentiation of MEL and CY action observed in tumours with large single doses of MISO. However, a chronic low dosing schedule of MISO did not affect the plasma half-life of either cytotoxic drug, although a significant potentiation of each drug in combination with a chronic MISO dose has been obtained in some tumours. These results suggest that potentiation of chemotherapeutic drug action by MISO in the clinical situation is unlikely to be due to changes in drug pharmacokinetics.

### Introduction

The 2-nitroimidazole radiosensitizer misonidasole (MISO) can significantly enhance the response of tumours to several chemotherapeutic drugs particularly at high doses (see review by McNally (1982)). Although several mechanisms have been postulated (Brown, 1982) the nature of the main ones responsible for this enhancement remain unclear. One possible explanation is that the radiosensitizer may alter the pharmacokinetics of the cytotoxic drug possibly in a way which would effectively increase the tissue exposure to the drug.

We have therefore investigated the effects of MISO on the pharmacokinetics of melphalan (MEL) and cyclophosphamide (CY) in order to try and determine the extent to which alteration in drug pharmacokinetics might contribute to the enhancement observed when a large single dose of MISO is given in combination with these agents. In addition, we have investigated the effect of chronic low doses of MISO given in a regime which more closely simulates human pharmacokinetics.

### Materials and methods

### *Mice*

WHT/GyfBSVS male mice between 3 and 4 months old, maintained in Category 4 specific pathogen-free conditions were used in all procedures. In two of the CY experiments female WHT mice (ex breeders) between 3 and 6 months old were used. They were not used in later experiments because it was subsequently found that older WHT mice suffer from a congenital kidney dysfunction.

### *Measurement of melphalan plasma levels*

Blood was obtained from the carotid artery after decapitation. Blood samples were collected in heparinised tubes, immediately cooled on ice and then centrifuged as soon as possible at 4°C. An aliquot of plasma was taken, frozen rapidly and stored at −20°C for subsequent analysis. Plasma was deproteinised with 2 volumes of methanol, cooled to −70°C to aid precipitation, centrifuged and the supernatant analysed for MEL by high performance liquid chromatography (HPLC). Chromatography was carried out using an LDC constametric pump, Waters WISP Autosampler, a Hypersil 5 ODS column and a Waters 441 uV detector operating at 254 nm; the results were presented and calculated on a Waters 730 data module calibrated against plasma samples containing a known concentration of MEL. The standard solution of MEL was prepared in methanol containing 2% acetic acid (Chang *et al.*, 1978). The eluant was 63% methanol; 5 mM heptane sulphonic acid, 2 mM% dibutylamine, 10 mM acetic acid, pumped at 2 ml min$^{-1}$.

### *Measurement of cyclophosphamide plasma levels*

An estimate of the concentration of active metabolites of CY in the blood at varying times after treatment was obtained using a modification of the tissue culture cytotoxicity assay first outlined

Correspondence: M. Hinchliffe
Received 4 May 1983; accepted 16 June 1983

© The Macmillan Press Ltd., 1983

by Weaver *et al.* (1978) and developed in this laboratory by Begg & Smith (in preparation). Blood was taken from the mice at varying times after i.p. injection of 200 mg kg$^{-1}$ CY with or without MISO. The brachial artery was cut in lightly anaesthetised mice and blood drawn up into a heparinsed syringe. The mice were then sacrificed without regaining consciousness. Blood pooled from 4–5 mice was kept on ice until it could be centrifuged. The serum was then removed, sterilised by filtration and diluted 1:20 in complete tissue culture medium (alpha-MEM plus 10% foetal calf serum). Two ml of the diluted "activated" CY was then added to V79 Chinese hamster cells plated several hours previously in 5 cm diameter Petri dishes. After incubating with the serum for 15 h, the plates were rinsed twice with Hanks' balanced salt solution and 5 ml of fresh medium added. Cell survival was estimated by counting the resulting colonies after incubating for 7 days at 37°C. Control plates received diluted serum from untreated mice.

### *Radiosensitizers*

For the acute doses 800 mg kg$^{-1}$ (4 mmol kg$^{-1}$) MISO was dissolved in saline at a concentration such that a 40 g mouse would receive 1 ml, and injected i.p. In the chronic dose schedules 120 mg kg$^{-1}$ was injected i.p. followed by 30 mg kg$^{-1}$ every 20 min for 8 h in order to maintain a plasma concentration of 100 $\mu$g ml$^{-1}$ over this period. This simulated the long half-life of MISO in man. For the chronic doses the drug was diluted so that a 40 g mouse would receive 0.2 ml. The cytotoxic drug was given immediately after the last dose of MISO. Mice treated with the cytotoxic drug alone received corresponding volumes of saline in both chronic and acute dose regimes. The radiosensitizer SR-2508 was administered by i.v. injection at a dose of 1070 mg kg$^{-1}$ (5 mmol kg$^{-1}$) at a concentration such that a 40 g mouse would receive 0.4 ml.

### *Cytotoxic drugs*

MEL was first dissolved in 0.5 ml 2% HCl in ethanol, then further diluted in saline. For pharmacokinetic studies, 10 mg kg$^{-1}$ was injected i.p. at a concentration of 0.4 ml per 40 g mouse within 10 min of dissolution. CY at a dose of 200 mg kg$^{-1}$ was injected i.p. dissolved in saline. As with MEL the concentration was such that a 40 g mouse received 0.4 ml. Linear regression analysis was used to calculate the "best fit" lines through the pharmacokinetics data for MEL. Drug half-lives were calculated from these lines, the errors given representing $\pm$1 s.d.

### Results

Figure 1(a) shows the plasma concentrations of MEL as a function of time after i.p. injections, either alone or 15 min after MISO at a dose of 800 mg kg$^{-1}$. In this and subsequent figures for MEL each point represents an individual mouse. Open symbols represent MEL alone and closed symbols represent combination of MISO with MEL. Different shapes are used to represent separate experiments. Because MEL is rapidly hydrolysed in aqueous solution at room temperature, MISO was given before rather than at the same time as MEL in order for the MEL to be injected as quickly as possible. MISO affected the pharmocokinetics of MEL in that it appeared to cause an extension of the peak concentration and a subsequent decrease in the rate of plasma clearance. The MEL plasma half-life was extended from 21 (19.5–21.5) to 50 (44.5–56.5) min. When MISO was given an hour before MEL (Figure 1(b)) there was a reduced effect on the rate of plasma clearance, $T_{1/2}$ = 34 min (32–36), but the plasma concentration remained at its maximum level for up to 40 min. Both Figures 1(a) and 1(b) are composites of 2 separate experiments.

Figure 2 shows the rate of decay of "active" CY metabolites in plasma in the presence or absence of MISO given simultaneously with (Figure 2(a)), or 1 h before CY (Figure 2(b)). In this and subsequent figures for CY each point represents the effect of serum pooled from 4–5 mice. An indication of the concentration of active CY metabolites in the plasma at a given time after injection was obtained indirectly by determining the toxicity of serum to V79 cells as described above. There was no effect of serum from untreated mice. Again MISO appeared to affect the pharmocokinetics of CY. The displacement of the curves with MISO relative to the controls indicates a retention of active CY metabolites in the plasma. There was some inter-experimental variation in the degree of cell killing obtained, particularly in the first 30 min after injection when plasma CY concentrations were changing rapidly. However, the displacement of the curves at times beyond 30 min did not vary between experiments.

It is possible that the *in vitro* interaction of MISO metabolites with activated CY was responsible for the decrease in V79 cell survival seen for the combined treatment, rather than a retention of active CY metabolites in the plasma. Serum from mice receiving MISO alone was therefore combined with that from mice receiving CY alone and added to V79 cells *in vitro*. Blood samples from both MISO and CY treated mice were taken at the same times after the injection of each drug to ensure, as far as possible, that both

**Figure 1** Plasma levels of MEL in WHT mice after 10 mg kg$^{-1}$ MEL alone (○, △, □), or 10 mg kg$^{-1}$ MEL plus 800 mg kg$^{-1}$ MISO (●, ▲, ■). (a) MISO—15 min—MEL; (b) MISO—1 h—MEL. Different symbols refer to separate experiments, each point represents an individual mouse. In this, and subsequent figures for MEL, lines of "best fit" were determined by linear regression analysis. Points occuring before the plasma clearance phase commenced were omitted from the analysis.

**Figure 2** Survival of V79 cells after treatment with plasma from WHT mice receiving 200 mg kg$^{-1}$ CY along (○), or 200 mg kg$^{-1}$ CY plus 800 mg kg$^{-1}$ MISO (●). (a) MISO and CY given simultaneously; (b) MISO—1 h—Cy. Each point represents the mean surviving fraction from four replicate plates having received diluted serum pooled from 4–5 mice.

drugs had been metabolised to the same extent as if given simultaneously to the same mouse. The resulting survival curves are shown in Figure 3. There was no effect of mixing "activated" MISO with "activated" CY *in vitro*. In the same experiment MISO and CY were given together to a group of mice and Figure 3 shows that, as before (Figure 2) the clearance of "active" metabolites appeared to be delayed.

A large single dose of MISO (800 mg kg$^{-1}$, 4 mmol kg$^{-1}$) administered to mice at room temperatures caused a reduction in rectal temperature of ~5°C (Figure 4(a)). This lowered temperature was reached within an hour of injection and was maintained for a further 4 h. The combination of this dose of MISO with CY (200 mg kg$^{-1}$ injected 60 min after MISO) caused a further reduction in temperature of 3–4°C (total

**Figure 3** Survival of V79 cells after treatment with plasma from WHT mice receiving 200 mg kg$^{-1}$ CY alone (○), 200 mg kg$^{-1}$ CY plus 800 mg kg$^{-1}$ MISO given simultaneously (●), 200 mg kg$^{-1}$ CY or 800 mg kg$^{-1}$ MISO and mixed *in vitro* (▲), (see text) or 800 mg kg$^{-1}$. MISO alone (■). Each point represents the mean surviving fraction from 4 replicate plates having received diluted serum pooled from 4–5 mice.

reduction, 9°C). This was maintained for at least 5 h and still appeared to be falling at the end of this period. Combination of MEL with MISO caused a total temperature reduction of 6°C (data not shown). If the mice were maintained at an ambient temperature of 37°C there was no significant fall in rectal temperature (Figure 4(b)) due to the MISO. However, the combination of MISO and CY then proved toxic to these mice. Mice receiving this combined treatment died between 1 and 4 h after receiving MISO, so that it was not possible to make pharmacokinetic measurements.

The effect of MISO on MEL pharmacokinetics was investigated when the mice were maintained in an ambient temperature of 37°C (Figure 5). This combination proved less toxic and the mice survived the duration of the experiment. Rectal temperatures were measured throughout the experiment and were not observed to drop with either MEL alone or the combination treatment. A change in MEL pharmacokinetics was still observed (Figure 5), although it was reduced compared to that at room temperature. MISO still appeared to prolong the peak plasma concentration at this temperature, but only a very small increase in the plasma half-life was observed from $21 \pm 1.5$ min to $27 \pm 2$ min, which may not be significant.

The nitroimidazole radiosensitizer SR-2508, unlike MISO, is not metabolised in the mouse (Workman & Brown 1981) and does not reduce body temperature; however, it has been found to potentiate the action of CY in the RIF-1 tumour (Law *et al.*, 1981). We therefore investigated the effect of SR-2508 on MEL pharmacokinetics. A dose of 1070 mg kg$^{-1}$ (5 mmol kg$^{-1}$) SR-2508 had no effect on the plasma half-life of MEL when injected 30 min before MEL (Figure 6). SR-2508 was injected 30 min rather than an hour before MEL, because of its shorter half-life in the mouse of 45–50 min for a dose of 5 mmol kg$^{-1}$ compared to 80–100 min for an equivalent MISO dose.

The effect of a chronic dosing schedule of MISO was also examined. A plasma concentration of 100 $\mu$g ml$^{-1}$ (0.5 mM) MISO was maintained for 8 h prior to giving the cytotoxic drug in an attempt to simulate the extended plasma half-life of MISO in man compared to that in mice. This is close to the plasma level which would be reached in man during

**Figure 4** Mouse rectal temperatures after 200 mg kg$^{-1}$ CY alone (-·-·-·-), 800 mg kg$^{-1}$ MISO—1 h—CY (----) or, no treatment (——). (a) at room temperature (20°C); (b) at 37°C. Each line represents one mouse.

Figure 5 Plasma levels of MEL in WHT mice maintained at 37°C after $10\,mg\,kg^{-1}$ MEL alone (○, □) or $10\,mg\,kg^{-1}$ MEL plus $800\,mg\,kg^{-1}$ MISO (●, ■). Circles and squares represent 2 separate experiments, each point represents an individual mouse. Lines of "best fit" were obtained by linear regression analysis, as previously described.

Figure 7 Plasma levels of MEL in WHT mice receiving $10\,mg\,kg^{-1}$ MEL immediately after an 8 h chronic dose of saline (○), or of MISO (●). Each point represents one mouse. The line of "best fit" was obtained by linear regression analysis, as described previously.

Figure 6 Plasma levels of MEL in WHT mice after $10\,mg\,kg^{-1}$ MEL only (△) or $1040\,mg\,kg^{-1}$ SR-2508 30 min before $10\,mg\,kg^{-1}$ MEL (▲). Each point represents an individual mouse. The "line of best fit" was obtained by linear regression analysis, as previously described.

a standard 6 fraction regime where $2\,g\,m^{-2}$ were administered each time.

The chronic dose schedule had no effect on the mouse body temperature and the plasma half-life of MEL was not altered when a dose of $10\,mg\,kg^{-1}$ was given at the end of the chronic MISO dosing (Figure 7). Similarly, the amount of active CY metabolites was not affected when a dose of $200\,mg\,kg^{-1}$ was given with the last MISO injection (Figure 8).

### Discussion

A large single dose of MISO ($800\,mg\,kg^{-1}$) had a considerable effect on the pharmacokinetics of MEL (Figures 1(a) and (b)), prolonging the peak concentration of MEL and delaying its subsequent plasma clearance. The effect varied according to the interval between the administration of the two drugs. When MISO was given 15 min before MEL (Figure 1(a)) there was a pronounced delay in the plasma clearance rate, and the MEL $T_{1/2}$ was increased from 20 (19.5–21.5) to 50 (44.5–56.5) min. However, when MISO was given an hour before MEL (Figure 1(b)), which is the usual interval used

**Figure 8** Survivial of V79 cells after treatment with plasma from WHT mice receiving $200 mg kg^{-1}$ CY immediately after an 8 h chronic dose of saline (○, □), or of MISO (●, ■). Each point represents the mean surviving fraction from four replicate plates having received diluted serum pooled from 4–5 mice.

in our tumour studies (McNally *et al.*, 1983) there was a reduced effect on the rate of plasma clearance, $T_{1/2} = 34$ min (32–36) but this was compensated by an increased retention of the peak plasma concentration. In both instances, the combined effects resulted in similar raised levels of MEL in the plasma at any given time after injection.

The results of the bioassay for CY indicate that MISO also delayed the clearance of active CY metabolites from the plasma (Figure 2). There was no noticeable difference between giving the MISO at the same time as CY (Figure 2(a)) or 1 h before (Figure 2(b)). However, any difference at early times were unlikely to be detected with the assay used (see above). Using a similar assay, Tannock (1980) has demonstrated retention of active CY metabolites in the serum of C3H mice after receiving $1000 mg kg^{-1}$ ($5 mmol kg^{-1}$) MISO. An increase in the plasma half-life of MEL with a large single dose of MISO has been reported by several other investigators (Stephens *et al.*, 1981; Clutterbuck, 1982; Lee & Workman, 1983). Similar effects have also been observed with BCNU (Tannock, 1980) and CCNU (Lee & Workman, 1983).

The same large dose of MISO as used in the present studies gave enhancement ratios (ERs) of 2.7. and 1.8 for MEL and CY respectively in the WHFIB tumour using a cell cloning assay (McNally *et al.*, 1983). It is possible that an alteration of drug pharmacokinetics could account for a significant proportion of this chemosensitization observed with large single doses of MISO. The existence of a large pharmacokinetic component of the interaction has previously been disputed on the grounds that it would not account for the large "therapeutic gains" observed by ourselves and other investigators (McNally, 1982) as more drug will be made available to both tumours and normal tissues. However, it is possible that prolonged exposure would lead to more drug being available to the tumour without a corresponding increase in normal tissue concentration. For example, Lee & Workman (1983) observed increased concentrations of CCNU in tumours relative to normal tissues, when combined with large doses of MISO.

Two possible mechanisms whereby MISO could affect drug pharmacokinetics are a reduction in mouse core temperature and/or inhibition of drug metabolism by MISO.

*Reduction in core temperature*

A dose of $800 mg kg^{-1}$ ($4 mol kg^{-1}$) MISO caused a reduction in body temperature of about 5°C which was maintained for 4 h (Figure 4(a)), well beyond the longest time at which drug plasma concentrations were measured. Similar temperature effects have been reported by Law *et al.* (1981) and Twentyman & Workman (1982). The latter investigators have shown that this reduced temperature persists for 10–12 h. In our experiments addition of $200 mg kg^{-1}$ CY 60 min after MISO further reduced mouse rectal temperature by about 3°C (total reduction ~9°C), whilst addition of $10 mg kg^{-1}$ MEL caused a total reduction of 6°C (results not shown).

The difference in MEL pharmacokinetics when given 15 or 60 min after MISO (Figures 1(a) and 1(b)) is difficult to explain. However it may be related to differences in the reduction of body temperature due to MISO at the two times; at 15 min the temperature had only fallen by 2°C, whereas the total reduction by 6°C was complete at 60 min. This increased reduction in temperature at 60 min may result in a decrease in the apparent volume of distribution which could be expected to cause either an increase in or extension of the peak plasma concentration of MEL. However, the same extension in the peak MEL concentrations was observed when the mice were maintained at 37°C (Figure 5), so that while there was no decrease in the rate of plasma clearance at this temperature, a significant increase in the MEL "area under the curve" was still obtained. Clearly a reduction in body temperature is not a sufficient explanation for the alteration of MEL pharmocokinetics due to MISO. Lee & Workman (1983) have also demonstrated changes in drug pharmacokinetics (in this case with CCNU) using smaller doses of MISO which did not affect body temperature.

The decomposition of MEL in plasma by hydrolysis has been shown to be extremely temperature dependent (Evans *et al.,* 1982), We measured minimal hydrolysis of MEL in mouse plasma *in vitro* at 29°C, whereas at 37°C the half-life due to hydrolysis was 210 min (Hinchliffe & Stratford, unpublished observations). The plasma clearance of MEL *in vivo* occurs with a half-life of 21 min (Figure 1(a)) and we have calculated that even if hydrolysis was completely arrested due to the reduction in body temperature caused by MISO, the half-life would only be increased by $\sim$2 min, whereas a half life of 34 min was observed when MISO was given 60 min before MEL. Clearly, changes in hydrolysis can only account for a small component of the changed MEL pharmacokinetics due to MISO.

These results suggest that reduction in body temperature can only partially account for the changes in MEL pharmacokinetics observed in combination with MISO. It was not possible to determine the effect of MISO on CY pharmacokinetics in mice held at 37°C, nevertheless we believe the same conclusion is probably true.

*Metabolic inhibition*

Workman *et al.* (1983) have shown that MISO inhibits hepatic drug-metabolising enzymes. MISO is itself metabolised by these enzymes to form desmethyl misonidazole (Ro-05-9963) (Shoemaker *et al.,* 1982). They have, therefore, suggested that MISO may delay the clearance of chemotherapeutic drugs by competitively inhibiting their metabolism (Workman *et al.,* 1983; Lee & Workman, 1983). This is supported by their findings that the cytotoxic action of CCNU and chlorambucil can be potentiated by inhibiting their metabolism with the drug SKF 525A (Workman & Twentyman, 1982). The anaesthetics Saffan and Sagatal have also been found to potentiate tumour cell killing by MEL (Peacock & Stephens, 1978; Peacock *et al.,* 1980). These agents are unrelated to MISO but are also metabolised by liver microsomal enzymes. The effect was unrelated to a body temperature reduction due to these drugs as the mice were maintained at 37°C.

The nitroimidazole radiosensitizer SR-2508 is metabolised to a much smaller extent than MISO in the mouse and does not reduce the body temperature (Workman & Brown, 1981). We have found that a dose of 5 mmol $kg^{-1}$ SR-2508 does not affect the plasma half-life of MEL (Figure 6) and does not potentiate the action of MEL in the WHFIB tumour (Hinchliffe, unpublished observations). This would tend to support the idea that MISO may act by competitively inhibiting drug metabolism. However, Law *et al.* (1981) have shown that SR-2508 potentiates the action of CY in the RIF-1 tumour.

In contrast the o-demethylation product of MISO, Ro-05-9963 is not appreciably metabolised in the mouse (Workman, 1980). However, we have found that it increased the plasma half-life of MEL in WHT mice and potentiated the action of MEL and CY in the WHFIB tumour (Hinchliffe, unpublished observations).

Our results indicate that the increase in MEL area under the curve results from both an extension of the peak plasma concentration and a subsequent reduction in the plasma clearance rate. Whilst metabolic inhibition would explain the delay in plasma clearance, it cannot so easily explain the prolonged peak plasma levels obtained. Moreover there is little evidence that MEL is metabolised to any appreciable extent (Furner & Brown, 1980; Evans *et al.,* 1982). As a result we feel that metabolic inhibition of plasma clearance by MISO is not a major factor in the case of MEL. The extension of peak levels would instead tend to indicate an effect on the initial distribution of the drug. It is possible that either the initial absorption of MEL from the peritoneum and/or its subsequent distribution into tissues may be impeded by MISO, possibly due to interference with the active transport of MEL across membranes (Vistica *et al.,* 1977).

*Chronic doses*

The use of large single doses of MISO in mice does not provide an adequate model with which to predict the effects of combining radio-sensitizers with chemotherapeutic drugs in the clinic because the half-life of MISO in man is much longer ($\sim$8 h) than in the mouse (40–120 min), and much smaller doses are used in man. We have attempted to simulate human pharmacokinetics of MISO by maintaining a plasma concentration of 100 $\mu g\,ml^{-1}$ (0.5 mM) for 8 h. This plasma level is within the clinical range and is close to that obtained in each fraction of a 6 fraction regime.

This chronic dose of MISO did not affect the pharmacokinetics of MEL when the cytotoxic drug was given at the end of the 8 h chronic dose period (Figure 7). Our tumour results are puzzling in that they appear to vary according to the method of assay. Large enhancement ratios (ERs) were obtained in the WHFIB tumour for both MEL and CY when an *in vitro* excision assay was used; however, no enhancement was observed using regrowth delay (McNally *et al.*, 1983) although there was enhancement of growth delay in another tumour, SaF. Conflicting results have been obtained by other investigators using similar dose regimes. Large tumour ERs have been observed by Brown & Hirst (1982) in the Rif-1 tumour, whereas Twentyman & Workman (1983) were unable to demonstrate any enhancement in the same tumour unless they extended the chronic dose schedule to 16h; also Randhawa (personal communication) has been unable to demonstrate any enhancement with a chronic MISO dose in a different tumour.

In view of the variability in results with chronic MISO doses further studies are required in order to determine if pharmacokinetic changes are associated with drug potentiation for chronic MISO doses, although there are suggestions that potentiation may occur without a pharmacokinetic effect in some tumours.

In spite of these variable results in different tumour systems, our observation that chronic MISO dosing does not affect MEL pharmokinetics suggests that in man there should be no effect of MISO on drug pharmacokinetics. However, Twentyman & Workman (1983) have suggested that a 16h chronic MISO dosing schedule is necessary in the mouse to simulate the human situation. It is possible that this extended exposure may have an effect on drug pharmacokinetics.

In conclusion, we have shown that a large single dose of MISO alters the pharmacokinetics of CY and MEL in mice. The mechanism for this pharmacokinetic effect remains unclear, but is probably in part related to the MISO induced reduction in body temperature. In the case of CY, MISO may also act by competitively inhibiting its metabolism, although this probably does not happen with MEL. We have found no effect of chronic low doses of MISO on CY or MEL pharmacokinetics, suggesting that altered drug pharmacokinetics may not be important in the clinical combination of MISO with chemotherapeutic drugs.

We are grateful to Ward-Blenkinsop (Bracknell) for supplying cyclophosphamide, the Wellcome Foundation for supplying melphalan and Roche Products, Ltd. (Welwyn) for supplying misonidazole, and Dr. P. Workman for supplying SR-2508. We are especially grateful to Mrs, J. de Ronde for excellent technical assistance and invaluable discussions and Mr. A. Minchinton for HPLC measurements of melphalan levels. We wish to thank the Cancer Research Campaign for financial support.

### References

BROWN, J.M. (1982). The mechanisms of cytotoxicity and chemosensitization by misonidazole and other nitroimidazoles. *Int. J. Radiat. Oncol. Biol. Phys.*, **8**, 675.

BROWN, J.M. & HIRST, D.G. (1982). Effect of clinical levels misonidazole on the response of tumour and normal tissues in the mouse to alkylating agents. *Br. J. Cancer*, **45**, 700.

CHANG, S.Y., ALBERTS, D.S., MELNICK, L.R., WALSON, P.D. & SALMON, S.E. (1978). High-pressure liquid chromatographic analysis of melphalan in plasma. *J. Pharm. Sci.*, **67**, 679.

CLUTTERBUCK, R.D., MILLAR, J.L. & MCELWAIN, T.J. (1982). Misonidazole enhancement of the action of BCNU and Melphalan against human melanoma xenografts. *Am. J. Clin. Oncol.*, **5**, 73.

COLVIN, M. (1978). A review of the pharmacology and clinical use of cyclophosphamide. In *Clinical Pharmacology of Anti-Neoplastic Drugs* p. 245. (Ed. Pinedo) Elscvier/North Holland Biomedical Press: Amsterdam.

EVANS, T.L., CHANG, S.Y., ALBERTS, D.S., SIPES, I.G. & BRENDEL, K. (1982). *In vitro* degradation of L-Phenylalanine Mustard (L-PAM). *Cancer Chemother. Pharmacol.*, **8**, 175.

FURNER, R.L. & BROWN, R.K. (1980). L-phenylalanine mustard (L-PAM) The first 25 years. *Cancer Treat. Rep.*, **64**, 559.

LAW, M.P., HIRST, D.G. & BROWN, J.M. (1981). Enhancing effect of misonidazole on the response of the Rif-1 tumour to cyclophosphamide. *Br. J. Cancer*, **44**, 208.

LEE, F.Y.F. & WORKMAN, P. (1983). Modification of CCNU pharmacokinetics by misonidazole—A major mechanism of chemosensitisation in mice. *Br. J. Cancer*, **47**, 659.

MCNALLY, N.J. (1982). Enhancement of chemotherapy agents. *Int. J. Radiat. Oncol. Biol. Phys.*, **8**, 593.

MCNALLY, N.J., HINCHLIFFE, M. & DE RONDE, J.S. (1983). Effects of chronic low doses of misonidazole on response of tumour and white blood cells in the mouse to alkylating agents. *Br. J. Cancer*, **48**, 271.

PEACOCK, J.H., JOINER, M.C. & STEPHENS, T.C. (1980). Modification of tumour cell kill by anaesthetics. *Br. J. Cancer*, **41**, (Suppl. IV) 311.

PEACOCK, J.H. & STEPHENS, T.C. (1978). Influence of anaesthetics on tumour cell kill and repopulation in B16 melanoma treated with melphalan *Br. J. Cancer*, **38**, 725.

SHOEMAKER, D.D., MCMANUS, M.E., HOERANT, R. & STRONG, J.M. (1982). Studies on the O-demethylation of misonidazole by rat liver microsomes. *Cancer Treat. Rep.*, **66**, 1343.

STEPHENS, T.C., COURTENAY, V.D., MILLS, J., PEACOCK, J.H., ROSE, C.M. & SPOONER, D. (1981). Enhanced cell killing in Lewis lung carcinoma and a human pancreatic carcinoma xenograft by the combination of cytotoxic drugs and misonidazole. *Br. J. Cancer*, **43**, 451.

TANNOCK, I.F. (1980). *In vivo* interaction of anti-cancer drugs with misonidazole or metronidazole: cyclophosphamide and BCNU. *Br. J. Cancer*, **42**, 871.

TWENTYMAN, P.R. & WORKMAN, P. (1982). Effect of misonidazole or metronidazole pretreatment on the response of the Rif-1 mouse sarcoma to melphalan, cyclophosphamide, chlorambucil and CCNU. *Br. J. Cancer*, **45**, 447.

TWENTYMAN, P.R., WORKMAN, P. (1983). An investigation of the possibility of chemosensitization by clinically achievable concentrations of misonidazole. *Br. J. Cancer*, **47**, 187.

VISTICA, D.T., TOAL, J.N. & RABINOVITZ, M. (1977). Amino acids affecting melphalan transport and cytotoxicity in cultered L1210 cells. *Proc. Am. Ass. Cancer Res.*, **18**, 26.

WEAVER, F.A., TORKELSON, A.R. ZYGMUNT, W.A. & BROWDER, H.P. (1978). Tissue culture cytotoxicity assay for cyclophosphamide metabolites in rat body fluids. *J. Am. Sci.*, **67**, 1009.

WORKMAN, P. (1980). Pharmacokinetics of hypoxic cell radiosensitizers. *Cancer Clin. Trials*, **3**, 237.

WORKMAN, P. & BROWN, J.M. (1981). Structure-pharmaco-kinetic relationships for misonidazole analogues in mice. *Cancer Chemother. Pharmacol.*, **6**, 39.

WORKMAN, P. & TWENTYMAN, P.R. (1982). Structure/activity relationships for the enhancement by electron-affinic drugs of the anti-tumour effect of CCNU. *Br. J. Cancer*, **46**, 249.

WORKMAN, P., TWENTYMAN, P.R., LEE, F.Y.F. & WALTON, M.I. (1983). Drug metabolism and chemosensitization: Nitroimidazoles as inhibitors of drug metabolism. *Biochem. Pharmacol.* (In Press).